# Emotional processing in bullying: an event-related potential study

**DOI:** 10.1038/s41598-022-12120-9

**Published:** 2022-05-13

**Authors:** Gisella Bonilla-Santos, Carlos Gantiva, Alfredis González-Hernández, Tatiana Padilla-García, Jasmin Bonilla-Santos

**Affiliations:** 1 Department of Psychology, Universidad Surcolombiana, Neiva, Colombia; 2 Department of Psychology, Universidad Cooperativa de Colombia, Calle 11 No 1-51, Neiva, 410010 Huila Colombia; 3 Department of Psychology, Universidad de los Andes, Bogotá, Colombia

**Keywords:** Physiology, Psychology

## Abstract

Bullying is a subtype of violence that leads to maladaptive behaviors and emotional responses, with implications for social competence, emotions, and empathy. The present study compared the time course of emotional processing in children who were involved in the dynamics of bullying (i.e., as victims, bullies, and observers) by evaluating event-related potentials [early posterior negativity and late positive potential (LPP)] in different brain regions during a passive visualization task that involved positive, neutral, and negative social pictures. High-density electroencephalograms were recorded in 45 children, 8–12 years old (*M* = 9.5 years, *SD* = 1.3), while they observed emotional and neutral social pictures that we selected from the International Affective Picture System. Late positive potential had higher amplitudes in the victim group, especially in posterior and anterior regions. In the central region, LPP was greater toward neutral social pictures in bullying victims. The greater amplitude of LPP in victims was observed during and after the stimulus. The results showed a consistent response with a higher intensity in response to emotional stimuli in the victim group, suggesting a tendency toward hypervigilance that could interfere with emotional regulation.

## Introduction

School bullying is a subtype of violence characterized by physical and psychological abuse. It is prolonged and perpetrated repeatedly by students or groups against other students or groups^1,2^. One in three children report having been bullied at some point in their lives^3^, 10–14% experience chronic bullying that lasts more than six months, 2–5% are bullies themselves, and a similar number are bullies/victims during childhood or adolescence^4^. Victims, bullies, victim-bullies (i.e., a victim subgroup that reacts with aggressive behavior), and observers who either reinforce or limit bullying are all participants in bullying situations^5^.

This phenomenon creates severe internalizing and externalizing symptoms in children and adolescents, including anxiety, depression, isolation, low self-esteem, suicidal behavior, psychosomatic symptoms, poor academic performance, and school dropout^6–12^. Abusers tend to experience difficulties in establishing affective and social relationships and following rules. They also present less self-control, insensitive traits, suicidal behavior, poor academic performance, school dropout, and a higher probability of engaging in criminal behavior^8,13–15^. Observers exhibit social and emotional maladjustment, characterized by increases in emotional isolation, anxiety, depression, hostility, and paranoia^16^.

Negative consequences of school bullying on social competence, empathy, and emotional life limit the proper psychological adjustment of individuals who are involved in bullying situations^17,18^. Additionally, experiencing bullying during childhood can modify the structure and function of the brain^19,20^ and neuroendocrine system, mainly the hypothalamus, which regulates stress^21^, thereby affecting the ability to process affective information^22^.

Despite the existence of several studies of school bullying and its consequences on emotional life in children, information about the underlying mechanisms of emotional processing in children that are linked to bullying from a neurophysiological perspective is still limited. Event-related potentials (ERPs) are used to study neural responses to emotional stimuli. In ERP research that investigates emotional imagery, attention to emotional stimuli is recorded more frequently than attention to neutral stimuli, commonly indicated by two ERP waves: early posterior negativity (EPN; i.e., an indicator of motivated selective attention) and late positive potential (LPP; i.e., an indicator of the focus of attention on important events)^23–25^.

The EPN component is involved in early stages of emotional processing. The EPN component amplitude is the consequence of a relative increase in negativity at temporo-occipital electrodes between 150 and 400 ms after stimulus onset^26^. It is considered an indicator of the processing of arousal when exposed to images or written words with emotional content^27,28^. Early posterior negativity indicates the degree of visual attention and the early encoding of affective discrimination in positive and negative emotions compared with neutral emotions. Studies have reported increases in EPN measures in response to angry expressions compared with happy faces in typically developing children^26^. The amplitude of EPN is influenced by age. Thus, in young children, it is topologically more extensive and has a much earlier onset (i.e., shorter latency) compared with older children and adults^29^. Nevertheless, the amplitude of this component appears to interact with the size and composition of stimuli^25,30^.

Studies of LPP have reported that emotional processing during childhood generates greater activation in the posterior region of the brain, and this activity extends to central and anterior regions during the maturation process^31,32^. Children with neurotypical development exhibit higher LPP amplitudes in response to pleasant and unpleasant stimuli compared with responses to neutral stimuli^32^. However, a higher LPP amplitude has been reported when individuals are exposed to unpleasant stimuli^33^, which could indicate negative attentional bias during and after the stimuli are presented^34^. Similarly, a higher LPP amplitude has been associated with anxiety and fearful behavior^32,35^.

Studies of emotional processing in children who were involved in school bullying indicated that bullying is a predictor of lifelong emotional problems^36^ and that negative emotions mediate the effects of victimization between peers with regard to the perpetration of harassment^37^. However, most studies in this field were based on self-reports. Therefore, information on differences in underlying psychophysiological mechanisms of emotion processing is limited. The present study compared the time course of emotional processing in children who were involved in the dynamics of bullying (i.e., as victims, bullies, and observers) by evaluating ERPs (i.e., EPN and LPP) in different brain regions using a passive visualization task that included positive, neutral, and negative social pictures.

Based on previous studies that found that negative childhood experiences that were associated with abuse situations influence the development of specific biases in emotional processing^38,39^, we hypothesized that there would be greater emotional reactivity over time, reflected by EPN and LPP components, in victims of bullying compared with bullies and observers.

## Methods

### Participants

Fifty-three children (24 females and 29 males) participated in the study. Eight participants were excluded from the analyses because of excessive movement artifacts during electroencephalographic (EEG) recordings. Therefore, 45 children (21 females), 8–12 years old (*M* = 9.51 years, *SD* = 1.35), were included in the study. The sample was divided into three groups based on scores on the School Bullying Questionnaire—Abbreviated and School Coexistence Questionnaire: victims (*n* = 19), bullies (*n* = 12), and observers (*n* = 14). Table 1 summarizes the basic demographic characteristics of the sample. The exclusion criteria were current medical or psychological treatment, intellectual disability, brain injury, epilepsy, and visual problems without correction.

**Table 1:** Demographic characteristics of the sample.

	Victims (*n* = 19)	Bullies (*n* = 12)	Observers (*n* = 14)	*χ*^*2*^ or *F*	*p*
Age (years) (mean [SD])	9.11 (1.32)	10.25 (1.28)	9.43 (1.28)	2.87	.068
Sex (% male)	57.9	75.0	28.6	5.87	.053
Sex (% female)	42.1	25.0	71.4		

*SD* standard deviation.

The study was approved by the Institutional Review Board of the Faculty of Health—Surcolombiana University (#5-008) and adhered to the tenets of the Declaration of Helsinki. All the participants provided written informed consent. Given that the participants were younger than 18 years of age, consent was also provided by their parents. The experimental procedures used in this study were approved by the Ethics Committee. Likewise, procedures were conducted in accordance with the relevant guidelines and regulations.

### Procedure

A passive visualization task was used. A total of 36 pictures were selected from the International Affective Picture System (IAPS)^40^, according to Colombian normative ratings^41^. Twelve pictures depicted positive social interactions (e.g., happy, loving, and smiling people; IAPS codes: 2071, 2347, 4628, 2395, 2151, 2511, 2274, 4622, 2155, 4626, 2158, and 2224), 12 pictures depicted neutral social interactions (e.g., neutrally looking people; IAPS codes: 2032, 2191, 2302, 2308, 2377, 2382, 2393, 2441, 2489, 2745.1, 6837, and 7493), and 12 pictures depicted negative social interactions (e.g., crying and suffering from pain; IAPS codes: 2095, 2375.1, 2683, 3005.1, 3101, 6315, 6520, 6555, 6563, 9163, 9413, and 9635.1). The pictures were presented on a 19-inch flat-screen monitor that was located approximately 60 cm from the subject. All the pictures were presented twice in four counterbalanced orders that consisted of 72 slides each, with the rule of not presenting the same picture from the social category consecutively. Pictures were presented for 1000 ms. A fixation mark ( +) was presented for 1000 ms before stimulus presentation. The intertrial interval varied randomly between 2000 and 4000 ms. The pictures were presented using E-Prime 2.0 software^42^.

### EEG/ERP recording and analysis

EEG data were collected from 64 channels based on the 10/20 system using the ActiveTwo BioSemi system (BioSemi, Amsterdam, The Netherlands), with a 256 Hz sampling rate. Electrode impedances did not exceed 20 kΩ. Electrooculograms were also recorded. All data processing was conducted using EEGLab^43^. The EEG data were first common average re-referenced. The resulting dataset was then bandpass filtered (from 0.1 to 30 Hz). The ERP data were segmented into epochs from 200 ms before stimulus onset to 1300 ms after stimulus onset. Correction for ocular artifacts was performed using the algorithm of Gratton^44^.

The EPN amplitude was evaluated as the area under the curve (AUC; PO4, POz, PO3, P3, P4, and Pz electrodes) using a time window between 175 and 275 ms^45^. LPP amplitudes were calculated as the AUC in two-time windows during picture presentation (early: 300–700 ms; late: 700–1000 ms;^32^) and one time window after picture presentation (1000–1300 ms^34^). The LPP was averaged in three regions: posterior (PO4, POz, PO3, P3, P4, and Pz), central (Cz, CPz, C4, C3, Cp4, and Cp3), and anterior (Fz, AFz, FC3, F3, FC4, and F4). Noisy epochs were detected and rejected when one or more channels exceeded a voltage threshold of ± 75 µV^32,46^. A total of 11.27% of the epochs were removed.

### Self-report measures

The Self-Assessment Manikin (SAM^47^) was used for affective evaluation of the pictures. The SAM is a pictorial nonverbal measure of emotion that consists of three affective 9-point scales: valence, arousal, and dominance. For the valence scale, the SAM ranges from a smiling, happy figure to a frowning, unhappy figure. For the arousal scale, the SAM ranges from a relaxed, sleepy figure with eyes close to an excited, wide-eyed figure. For the dominance scale, the SAM ranges from a very small figure that represents a feeling of being controlled to a very large figure that represents a feeling of being in control.

The School Bullying Questionnaire—Abbreviated^48^ was used to detect the risk of bullying in subjects between 8 and 18 years of age. The instrument consists of three scales. The first scale evaluates situations of bullying victimization (physical, verbal, social, and coercion), with a Cronbach’s alpha of .87. The second scale refers to bullying by respondents, with a Cronbach’s alpha of .83. The third scale explores anxiety, depression, and posttraumatic stress symptoms and effects on self-esteem, with a Cronbach’s alpha of .89.

The School Coexistence Questionnaire^49^ was used to identify school bullying by considering three types of participants (bully, victim, and observer) and different types of aggression (physical, social, and verbal). A validated version was used for the sample of children between 7 and 12 years of age.

The Childhood Depression Inventory^50^ is used to evaluate clinical indicators of depression in children and adolescents. The validated Spanish version consists of 27 items, each expressed in three sentences that convey depressive symptomatology with different frequencies. The content of the items covers most criteria for the diagnosis of childhood depression. The results of the test provide data on total depression and two additional scales (dysphoria and negative self-esteem)^51^.

The State-Trait Anxiety Inventory for Children^52^, adapted for the Spanish population, consists of two independent scales, one that evaluates state anxiety and one that evaluates trait anxiety. The state anxiety scale contains 20 items that evaluate a child’s level of anxiety at a given moment in time. The trait anxiety scale contains 20 items that evaluate how the child feels in general^53^.

### Statistical analysis

EPN amplitudes and SAM scores were analyzed using a 3 × 3 repeated-measures analysis of covariance (RM-ANCOVA), with group (victim, bully, observer) as the between-subjects factor and picture (positive, neutral, and negative) as the repeated measure. LPP amplitudes were analyzed separately for each brain region using a 3 (group) × 3 (picture type) × 3 (time window: early, late, and post-picture) RM-ANCOVA. When the assumption of sphericity was not met, Greenhouse–Geisser correction was applied to the degrees of freedom in all cases. Post hoc analyses of mean values were performed using paired multiple comparisons with Bonferroni correction. The level of significance was *p* < .05. All of the statistical analyses were performed using SPSS 20.0 software.

## Results

### Early posterior negativity

No significant main effects or interactions were found (all *p* > .08).

### Late positive potential in posterior region

Table 2 presents descriptive statistics for LPP amplitudes in the victim, bully, and observer groups for each region and time window of analysis. In the posterior region, LPP varied according to group (*F*_2,40_ = 12.82, *p* < .001, ηp^2^ = .39) and marginally varied according to time window (*F*_2,80_ = 3.31, *p* = .06, ηp^2^ = .07). Additionally, the ANCOVA revealed a significant group × time window interaction (*F*_4,80_ = 5.28, *p* = .005, ηp^2^ = .20).

**Table 2:** Means (SD) for LPP amplitudes (AUC) for positive, neutral, and negative pictures for the three regions (posterior, central, and anterior).

	Victims (*n* = 19)			Bullies (*n* = 12)			Observers (*n* = 14)		
	Early	Late	Post	Early	Late	Post	Early	Late	Post
**Posterior**									
Positive	1452.2 (521.5)	764.1 (282.3)	476.8 (196.7)	1122.5 (427.7)	534.9 (265.4)	349.6 (130.4)	830.0 (325.5)	428.7 (189.7)	291.0 (127.7)
Neutral	1463.4 (529.9)	640.6 (168.8)	386.5 (101.1)	1115.0 (566.5)	504.0 (254.2)	271.3 (107.9)	864.6 (304.9)	420.9 (140.0)	329.1 (147.7)
Negative	1749.1 (404.5)	1010.1 (259.5)	579.7 (191.7)	1190.2 (474.3)	695.9 (287.0)	385.5 (123.4)	986.9 (372.3)	590.9 (247.8)	351.5 (107.8)
**Central**									
Positive	715.4 (142.9)	334.5 (77.8)	296.5 (64.4)	698.4 (359.1)	296.2 (170.4)	225.5 (67.7)	708.7 (165.3)	306.7 (80.2)	247.4 (55.6)
Neutral	855.5 (211.3)	423.4 (188.5)	344.6 (122.9)	818.5 (331.8)	334.9 (130.7)	236.6 (65.8)	666.5 (236.9)	284.5 (84.2)	220.2 (57.5)
Negative	817.0 (196.9)	354.6 (62.7)	323.1 (93.1)	802.6 (363.7)	296.4 (133.3)	246.5 (72.2)	764.0 (240.4)	307.9 (102.3)	259.2 (87.7)
**Anterior**									
Positive	1152.3 (348.0)	611.7 (207.3)	412.4 (130.3)	956.6 (193.4)	427.8 (221.5)	286.3 (94.1)	827.1 (258.3)	441.8 (181.1)	321.3 (92.1)
Neutral	1139.1 (381.4)	479.6 (127.4)	299.1 (59.8)	972.6 (349.8)	441.9 (182.8)	250.8 (68.3)	816.7 (316.8)	424.5 (191.3)	322.2 (180.6)
Negative	1389.0 (300.5)	747.9 (175.8)	374.2 (86.2)	1066.5 (316.2)	573.4 (284.2)	330.2 (125.7)	916.2 (296.2)	538.6 (233.6)	280.6 (70.7)

LPP time windows are divided into early (300–700 ms), late (700–1000 ms), and post stimuli (1000–1300 ms).

*SD* Standard deviation, *AUC* Area under the curve, *LPP* Late positive potential.

**Figure 1:**
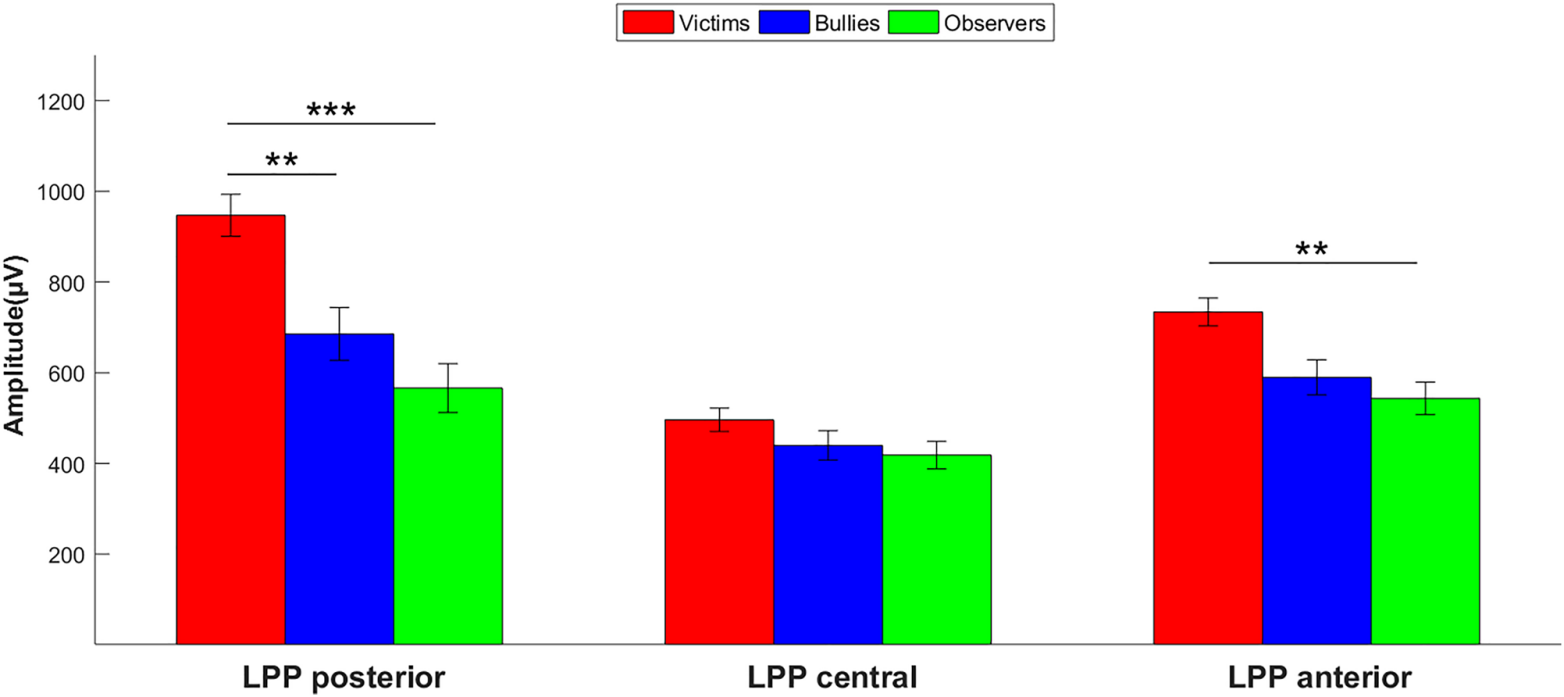
Amplitude of LPP for each group. Note: *p < .05. LPP: Late Positive Potential. Bars indicate ± 1 SEM.

**Figure 2:**
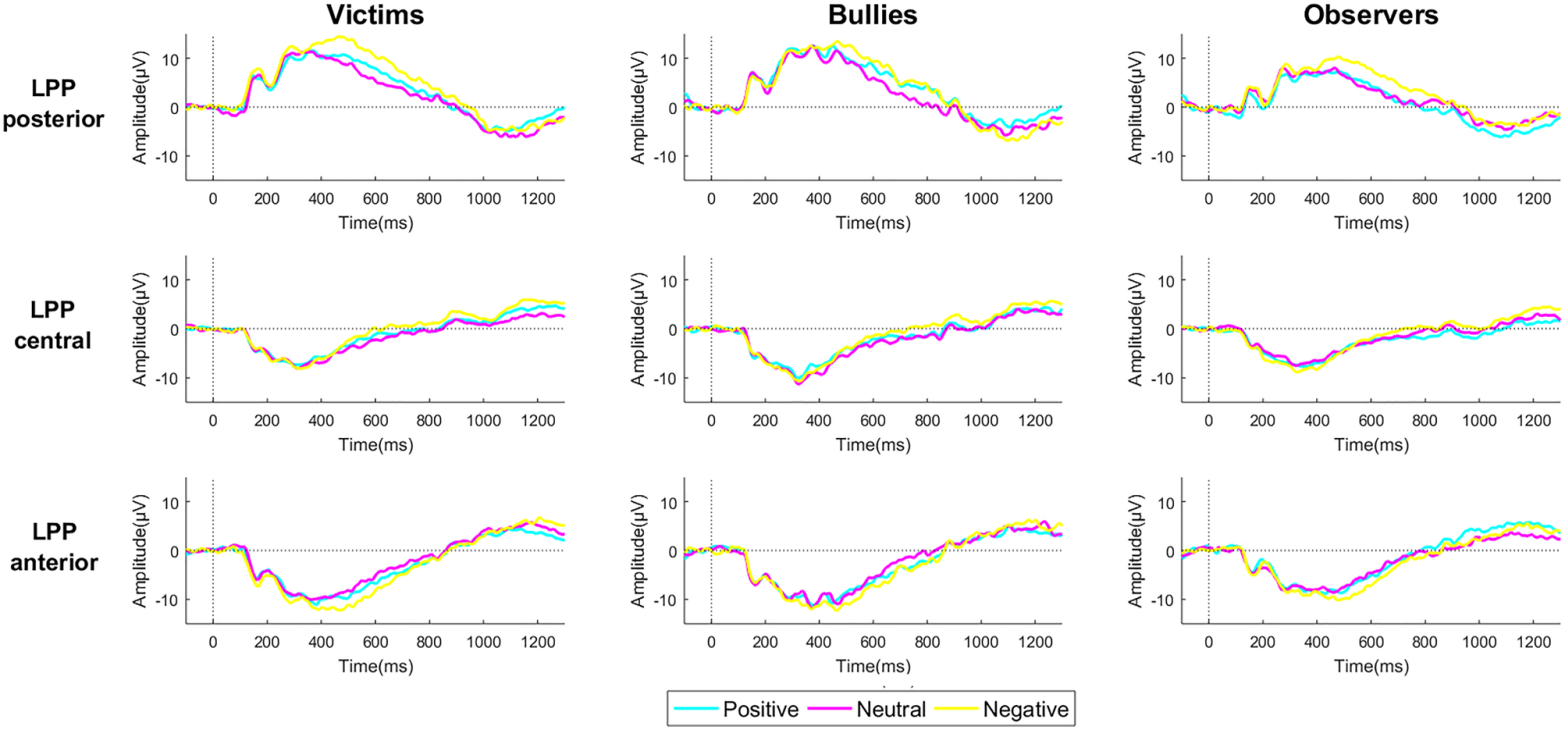
Grand average ERP LPP waveforms in the victim, bully, and observer groups in posterior, central, and anterior regions.

The post hoc analyses indicated that LPP amplitudes were greater for victims compared with bullies (*p* = .006, *d* = .74) and observers (*p* < .001, *d* = 1.13; Figs. 1 and 2). LPP amplitudes were higher in the early time window during picture presentation and progressively decreased to the time window after picture presentation (all *p* < .001, all *d* > 1.61). The significant group × time window interaction showed that victims had higher LPP amplitudes than bullies and observers in all time windows (all *p* < .021, all *d* > .59).

### Late positive potential in central region

In the central region, LPP varied according to time window (*F*_2,80_ = 5.12, *p* = 0.02, ηp^2^ = .11), with a significant group × picture type interaction (*F*_4,80_ = 2.82, *p* = 0.03, ηp^2^ = .12). LPP amplitudes were higher during the early time window during picture presentation and progressively decreased to the time window after picture presentation (all *p* < .001, all *d* > .76). The significant group × picture type interaction showed that victims had higher LPP amplitudes than observers in response to neutral pictures (*p* = .02, *d* = .60).

### Late positive potential in anterior region

In the anterior region, LPP varied according to time window (*F*_2,80_ = 13.52, *p* < .001, ηp^2^ = .25) and group (*F*_2,40_ = 7.10, *p* = .002, ηp^2^ = .26), with a significant group × time window interaction (*F*_4,80_ = 3.79, *p* = .01, ηp^2^ = .16). The post hoc analyses indicated that LPP amplitudes were higher in the early time window during picture presentation and progressively decreased to the time window after picture presentation (all *p* < .001, all *d* > 1.66). Victims had higher LPP amplitudes than observers (*p* = .003, *d* = .85) and marginally higher LPP amplitudes than bullies (*p* = .06, *d* = .61; Figs. 1 and 2).

The significant group × time window interaction showed that victims had higher LPP amplitudes than observers in the early time window (*p* = .003, *d* = .86) and late time window (*p* = .03, *d* = .55) during picture presentation. Victims had higher LPP amplitudes than bullies in the last time window after picture presentation (*p* = .003, *d* = .65).

### Subjective measures

The ANCOVA of the valence dimension revealed a significant main effect of group (*F*_2,40_ = 3.94, *p* = .02, ηp^2^ = .16). Bullies experienced lower levels of valence (i.e., more aversive) compared with victims (*p* = .02, *d* = .58). No other significant main effects or interactions were found.

Table 3 present the self-report scores on the School Bullying Questionnaire in the victim group were related to indicators of trait anxiety (*r* = .71, *p* = .001), fear (*r* = .69, *p* = .002), avoidance (*r* = .54, *p* = .01), dysphoria (*r* = .59, *p* = .004), and total scores on the Childhood Depression Inventory (*r* = .50, *p* = .01). Among bullies, scores on the negative self-esteem scale (*r* = .61, *p* = .04) and total scores on the Childhood Depression Inventory showed a positive relationship (*r* = .65, *p* = .02).

**Table 3:** Correlation between school bullying questionnaire scores and clinical scales.

Measure	Victims			Bullies			Observers		
	Me	IQR	*r*	Me	IQR	*r*	Me	IQR	*r*
TA	31.5	54	0.71**	31.0	27	0.29	26.5	25	0.06
SA	31.0	14	0.02	31.0	9	− 0.26	32.0	10	− 0.31
Fear	5.0	8	0.69**	5.0	4	0.41	4.0	5	− 0.50
Reassurance	7.0	9	− 0.03	7.0	6	− 0.51	7.5	4	− 0.31
Worry	6.0	9	0.28	6.0	6	0.25	5.0	6	0.13
Avoidance	4.5	9	0.54*	4.0	6	0.48	3.5	5	− 0.03
Dysphoria	60.0	81	0.59**	75.0	86	0.25	35.0	95	− 0.12
Self-esteem	50.0	82	0.37	60.0	91	0.61*	25.0	95	− 0.34
CDI	45.0	86	0.50*	75.0	92	0.65*	32.5	96	− 0.27

*TA* trait anxiety, *SA* state anxiety, *CDI* children's depression inventory.

*p < .05; **p < .001.

## Discussion

The present study compared the time course of emotional processing in children who were involved in the dynamics of bullying (i.e., as victims, bullies, and observers) by evaluating event-related potentials (i.e. EPN, LPP) in different brain regions during a passive visualization task that involved positive, neutral, and negative social pictures. Similar to other studies, we found that negative images elicited greater emotional activation and attentional engagement (i.e., higher LPP amplitude) compared with neutral and positive images^32,45,54^. Victims of bullying had higher EPN and LPP amplitudes, suggesting greater emotional activation and demand for attentional resources. Similar results (i.e., a greater LPP amplitude and increases in anxiety) were reported in studies that analyzed emotional responses in children who were exposed to domestic violence that was perpetrated by parents^55–57^.

The EPN component reflects early affective discrimination and the evaluation of salient stimuli^58^. In the present study, no significant differences were found in EPN amplitude between groups. Similar results were found in 5–8-year-old children, suggesting that the earlier and more automatic detection of emotional stimuli that is reflected by EPN could be evident until later in development^45,59^. Thus, the age of the children in this study could explain the absence of significant differences.

With regard to the effect of the images on the time course of emotional and attentional processes, the LPP distribution showed a dynamic electrocortical response in three temporal windows: early, late, and after presentation of the stimulus. Consistent with previous findings, was found permanent activation in the posterior region, mainly in the early window during presentation of the picture, which gradually decreased until the posterior temporal window of presentation of the picture^32,54^. The victims had higher amplitudes than bullies and observers at all time windows, which was in agreement with Fang et al. (2019), in which continuous activation indicates the permanence of specific biases in the emotional processing and states of hyperactivity^38^. The results suggested that aggression and exclusion among peers result in high attention and emotion neurophysiological indices.

In the central region, the LPP amplitude was higher in victims compared with observers for neutral images. The present findings differ from previous studies that reported greater activation only for unpleasant images. Children who are exposed to bullying may dedicate more attentional resources to neutral social stimuli, perhaps because neutral social stimuli can be interpreted as ambiguous and potentially harmful. Similar results were reported in previous studies with clinical infant samples who were exposed to different stress conditions and emotional distress^38^. These findings differ from previous studies that reported greater activation only for unpleasant images, which may suggest a tendency toward interpreting neutral social cues as threatening because of a state of reactive hypervigilance, which in turn would lead this group to less efficiently regulate emotional responses^60^.

Responses of the amygdala to emotional neutral images have been suggested to be exaggerated in patients with posttraumatic stress disorder^61,62^. Such responses could be interpreted as the hyperactivation of structures that are involved in responses to neutral stimuli, as a generalized threshold of threatening to non-threatening information processing. This finding suggests a tendency among victims to interpret neutral signals as threats because of a state of reactive hypervigilance that in turn leads victims to be less efficient in regulating emotional responses^63^.

The difference in the LPP activation response in the anterior region in the victim group was constant during stimulus presentation compared with bullies and observers. The results are generally consistent with previous studies that investigated LPP as a topographically dynamic response^60^; and identified regional patterns that reflect the development of connections between visual processing and attention networks in the frontal area^32,64^. This suggests sustained network activity in anterior–posterior regions, indicating bidirectional prefrontal-occipitoparietal modulation^59,65^.

## Conclusion

In conclusion, bullying is interpreted as a potential real-world threat that has an unpredictable time, intensity, frequency, and duration. Victims of school bullying are prone to experience high levels of anticipatory anxiety and depression^65^. As a result, they develop more reactive responses, creating a generalized state of hypervigilance and apprehension that can manifest as excessive worry and chronic distress^66^.

These responses are related to a neurophysiological marker of emotional and attentional modulation, such as LPP^7,67^. Differences in LPP were identified as a specific response in the victim group, this type of response may be related to connections between emotional processing and fearful behavior and anxiety states^54^. These finding broaden our understanding of the phenomenon of school bullying by identifying a possible neurophysiological vulnerability factor. Our findings may contribute to the design of prevention and intervention strategies that associate modulation of the LPP component with cognitive reappraisal, given its stability as a measure of emotional processing during development^32,57^.

The present study has limitations. One limitation of the present study was the relatively small sample size. Because we classified the bully, victim, and observer groups according to specific profiles, a stratified group analysis to evaluate possible sex differences was not possible. The profiles were identified using a self-report instrument. Given the social connotation of bullying, it is likely to create classification bias specifically for the bully profile. However, the instruments were applied in the school setting, which allowed an observation and confirmation process of profiles. Lastly, the number of pictures by categories may have influenced the results, however all the pictures were presented twice in four counterbalanced orders that consisted of 72 slides each. Future studies should explore interactions among sociodemographic, clinical, and personality factors within each profile (bullies, victims, and observers) that allow the assessment of LPP as a biomarker of attentional bias that is engaged in fear, anxiety, and depression.

## Data Availability

The datasets generated during the current study are available from the corresponding author on reasonable request.
